# Cardio-Oncology: A Myriad of Relationships Between Cardiovascular Disease and Cancer

**DOI:** 10.3389/fcvm.2022.727487

**Published:** 2022-03-17

**Authors:** Yinghui Wang, Yonggang Wang, Xiaorong Han, Jian Sun, Cheng Li, Binay Kumar Adhikari, Jin Zhang, Xiao Miao, Zhaoyang Chen

**Affiliations:** ^1^Department of Cardiovascular Centre, The First Hospital of Jilin University, Changchun, China; ^2^Department of Cardiology, Nepal APF Hospital, Kathmandu, Nepal; ^3^The Second Hospital of Jilin University, Changchun, China; ^4^Department of Cardiology, Union Hospital, Fujian Medical University, Fuzhou, China

**Keywords:** cardiology, oncology, cardiovascular disease, cancer, cardiotoxicity

## Abstract

Cardiovascular disease (CVD) and cancer are the leading causes of death worldwide. With an increasing number of the elderly population, and early cancer screening and treatment, the number of cancers cases are rising, while the mortality rate is decreasing. However, the number of cancer survivors is increasing yearly. With the prolonged life span of cancer patients, the adverse effects of anti-tumor therapy, especially CVD, have gained enormous attention. The incidence of cardiovascular events such as cardiac injury or cardiovascular toxicity is higher than malignant tumors' recurrence rate. Numerous clinical studies have also shifted their focus from the study of a single disease to the interdisciplinary study of oncology and cardiology. Previous studies have confirmed that anti-tumor therapy can cause CVD. Additionally, the treatment of CVD is also related to the tumors incidence. It is well established that the increased incidence of CVD in cancer patients is probably due to an unmodified unhealthy lifestyle among cancer survivors or cardiotoxicity caused by anti-cancer therapy. Nevertheless, some patients with CVD have a relatively increased cancer risk because CVD and malignant tumors are highly overlapping risk factors, including gender, age, hypertension, diabetes, hyperlipidemia, inflammation, and obesity. With advancements in the diagnosis and treatment, many patients simultaneously suffer from CVD and cancer, and most of them have a poor prognosis. Therefore, clinicians should understand the relationship between CVD and tumors, effectively identify the primary and secondary prevention for these diseases, and follow proper treatment methods.

## Introduction

Currently, cardiovascular disease (CVD) and cancer have the highest morbidity and mortality worldwide. They are closely related in terms of many factors, including risk factors, pathogenesis, and iatrogenic side effects. The lifetime risk of CVD in people >30 years old is close to 50%, causing ~17.3 million deaths worldwide each year (1). In 2020, ~19.3 million people were diagnosed with cancer globally, resulting in approximately 10 million deaths. It is worth noting that the first year of cancer is the period with the highest mortality from cardiovascular complications (2). Therefore, as a severe global public health threat, the relationship between CVD and cancer is actively being studied and updated. Various exogenous factors and genes have contributed to the onset of these diseases. Anti-cancer treatments have led to an increase in the incidence of CVD. Similarly, antihypertensive drugs (angiotensin converting enzyme inhibitors; ACEI) and aspirin affect the occurrence of different types of cancer. We summarized relevant studies and proposed that CVD and cancer are predisposing factors for each other. Starting from the pathogenesis, this article systematically summarizes the epidemiological status, points out the common occurrence and pathogenic mechanisms of various CVD and cancer, lists anti-cancer drugs, and discusses several cardiovascular side effects induced by anti-cancer therapy. Finally, we provide the latest strategies for the clinical management of such patients.

## Relationship Between Cancer and Cardiovascular Disease

### Common Risk Factors

CVD and cancer are multifactorial, with highly overlapping risk factors, including smoking, metabolic syndrome, radiation, age, air pollution, and environmental toxins (3). Recent studies have pointed out that CVD and tumors have direct mutual effects in addition to the above risk factors. CVD can increase the overall incidence of cancer. In patients with early-stage breast cancer, 59% of post-treatment recurrence and 60% of cancer-specific deaths are related to cardiovascular events (4, 5). Heart failure may be a risk factor for tumors by releasing particular heart failure-associated proteins, such as SERPINA3, into the bloodstream, leading to tumor development and growth, while elevated cardiac and inflammatory markers may indicate new cancers, according to an experimental study (6). Hypertension has a similar mechanism to tumors. Hypertension affects the arterial wall through oxidative stress and is related to cell canceration (7). Patients with hypertension have 2-fold higher risk cancer of normal people.

With normal blood pressure, and the incidence of malignant tumors increases with the increase of blood pressure. Active and effective antihypertensive treatment can prevent cardiovascular complications and improve the quality of life in cancer patients. Grossman et al. (8) further showed that high blood pressure increased the risk of cancer death by 23%. Myocardial infarction, as an acute rational stressor that accelerates breast cancer progression, accelerates tumor growth by activating systemic host response; meanwhile, bone marrow cells present an immunosuppressive state, accelerating the division of monocytes, and promoting tumor proliferation (8, 9). In addition, tumors promote the development of CVD. Tumors consume glucose in the body in a form other than insulin, leading to systemic insulin loss, and cardiac atrophy and heart failure, and possibly accelerating tumor progression (10). Moreover, many drugs and radiotherapy in cancer treatment are cardiotoxic, which aggravate the occurrence and development of heart failure, arrhythmia, coronary artery disease, hypertension and other CVD.

### Genetic Susceptibility

TET2 is the most common mutated gene associated with increased incidence and mortality due to CVD, which is also the first gene identified as an acquired mutation in individuals without hematological malignancies. Preclinical studies have shown that TET2-deficient cells can accelerate atherosclerosis in mice, promoting the release of IL-1β from macrophages; consequently, perpetuating vascular inflammation and inducing monocyte aggregation at the lesion site to aggravate inflammation (11). Hereditary/familial cardiomyopathy (CMY) is an autosomal dominant monogenic disease with no clear cardiac abnormality (12). Hypertrophic cardiomyopathy (HCM) has the highest incidence among CMY, followed by dilated cardiomyopathy (DCM), arrhythmogenic cardiomyopathy (ACM), and restrictive cardiomyopathy (13). In 2012, Truncations of titin (*TTN*) was first proposed as a DCM related gene, which encodes myotin in sarcomere (14). Truncating variants in the *TTN* gene (*TTNtv*), i.e., *TTN* trunk-frame-frame-mutation was detected in 25% of familial cardiomyopathy, 18% of sporadic cardiomyopathy, 10% of perinatal cardiomyopathy, and 25% of alcoholic cardiomyopathy. Patients with *TTNtv* had worse cardiac function (15). *TTNtv* carriers are likely to have cancer treatment-induced cardiomyopathy (CCM). Genetic susceptibility to DCM increases susceptibility to CCM (16). 90% of patients with CCM received anthracyclines. An animal study showed that mice with *TTNtv* showed left ventricular cardiomyocyte elongation and dysfunction after treatment with anthracycline (13). In addition, desmosomes, as the main structure of the connections between cells, inhibit cell motor ability. Mutations in desmosome genes have been detected in various cancers and ACM patients (13). In a study of cardiomyopathy induced by cancer treatment in children, the risk of CCM in Africa is higher than in Europe, possibly due to abnormal expression of putative homeodomain transcription factor 1 (PHTF1) and associated with long-term response to adriamycin therapy (17). Therefore, genetic screening provides guidance to identify patients at high risk for CCM and helps evaluate drugs for prevention and treatment and optimize the treatment of cancer and CVD.

### Clonal Hematopoiesis

Clonal hematopoiesis (CH) is defined as the clonal expansion of blood cells in the presence of somatic mutations and is an age-related biological state (18). The concept of clonal hematopoiesis of indeterminate potential (CHIP) was first introduced in 2015 as the presence of somatic mutations associated with hematologic malignancies in the blood or bone marrow of individuals with non-malignant hematologic diseases (19), involved in tumor development and CVD (20). CHIP is a biological state associated with aging that is virtually absent in children and its expression increases with age, mainly in the form of hematopoietic stem cell mutations. CHIP produces clonal white blood cells to populate peripheral blood, and individuals with 2–3 of these somatic mutations in a row are at increased risk of developing leukemia (21). Therefore, CHIP is considered a preclinical state for malignant blood disorders. Notably, CHIP alters innate immune cells to promote lymphoid malignancy and accelerates solid cancer progression by disrupting acquired immune cell homeostasis (22). In addition, chemotherapy promotes clonal expansion of specific mutations, leading to poor outcomes (23).

CHIP is an independent risk factor for CVD. CHIP increases the risk of atherogenesis and accelerates atherosclerosis and chronic cardiac insufficiency, leading to a poor prognosis for such patients (20). In 2017 Jaiswal et al. (24) enrolled 4,726 participants with coronary heart disease and 3,529 controls and confirmed that the presence of CHIP in peripheral blood cells can lead to a doubling of the risk of developing coronary artery disease. The study also found that the degree of coronary artery calcification and the incidence of coronary events were positively associated with CHIP (24). Atherosclerosis due to CHIP-associated mutations are primarily mediated by inflammation. The presence of CHIP-associated mutations in macrophages stimulates inflammation and changes the levels of inflammatory factors. Moreover, persistent chronic inflammatory state positively feeds back into somatic mutations leading to increased CHIP-associated mutations, promoting the development and progression of atherosclerosis (25–28). This finding also explains the role of CHIP in patients with valvular lesions, where the presence of CHIP has been shown to accelerate valve sclerosis in patients with aortic stenosis and often leads to a poor prognosis, and CHIP increases mortality even after aortic valve replacement (29, 30). Besides, 2021 Pascual et al. (31) observed that CHIP was common in patients with reduced left ventricular ejection fraction (LVEF) and was highly associated with accelerated heart failure progression regardless of etiology.

### Multiple Strike Theory

**Figure 1:**
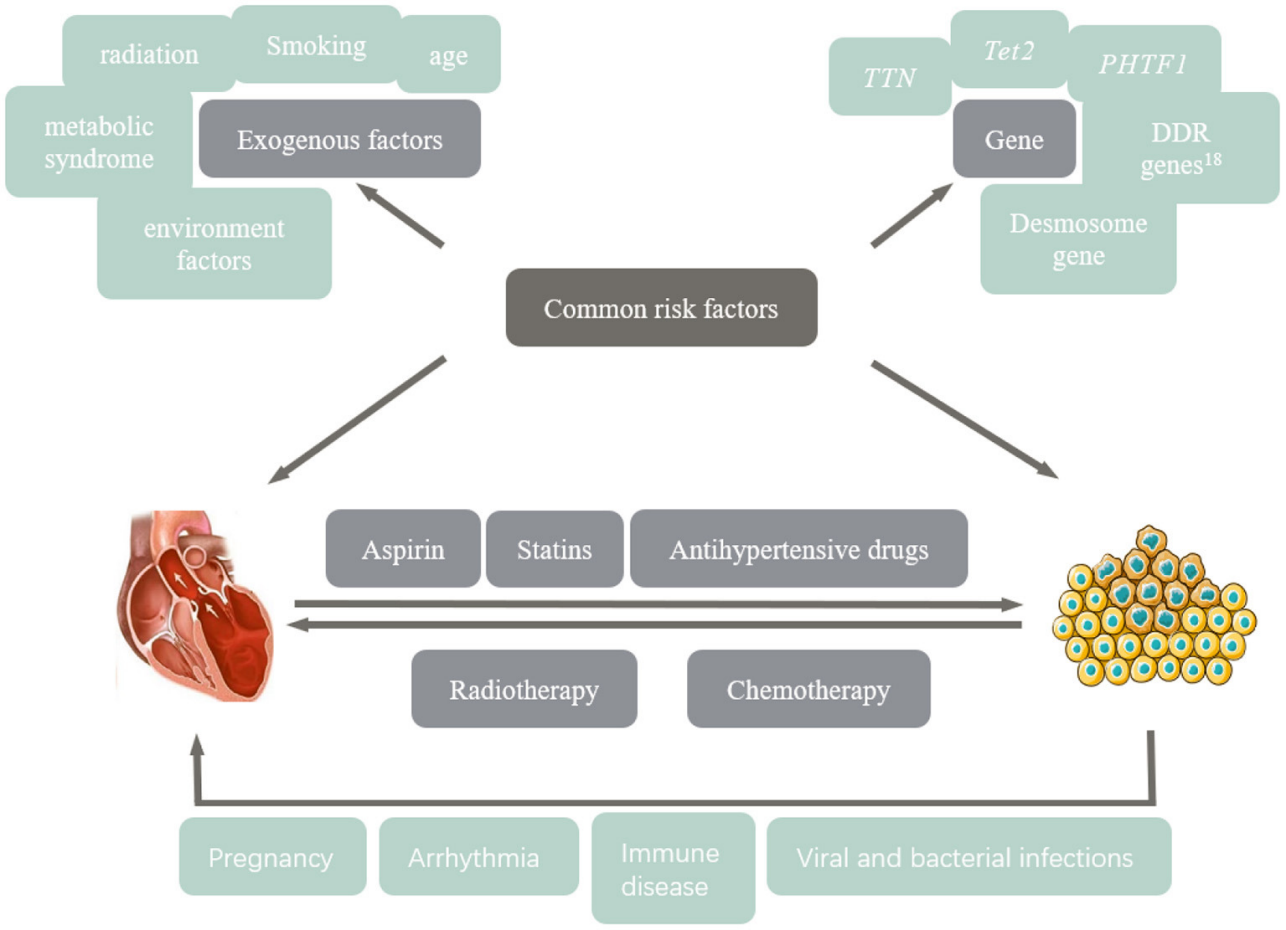
The multiple blow theory is that anti-tumor therapy leads to cardiotoxicity.

Multiple strike theory (Figure 1) is critical in the pathogenesis of cancer and CVD; the more the risk factors, the higher the incidence of disease. Children and adolescents with cancer have a good prognosis after antitumor therapy, while poor prognosis with cardiovascular side effects when the heart is stressed by pregnancy, hypertension, diabetes and hyperthyroidism (3).

First, tumors are associated with the pathogenesis of CVD, including the induction of exogenous factors, such as adverse lifestyle and endogenous factors, namely certain gene mutations, such as *TTNtv*, affecting the occurrence of DCM and CCM. Secondly, both anti-tumor therapy and treatment of CVD may further deteriorate cardiovascular function, cause tumor, and affect the prognosis. These common risk factors, genetic predisposition, therapeutic interventions, and the progressive involvement of certain diseases that stress the heart may contribute to the gradual development of cardiotoxicity during antitumor therapy.

## Commonly Used Drugs for Patients With Cardiovascular Disease May Affect the Occurrence of Cancer

### Aspirin

Aspirin is widely used in the primary and secondary prevention of CVD due to its effects on platelet aggregation, atherosclerotic thrombosis, and embolization. Platelet activation prevents tumor cells from elimination by an immune response and promotes their retention in endothelial cells and growth of metastatic cells, as well as contributes to angiogenesis, thereby promoting metastasis (32). Therefore, aspirin can slow the metastatic spread of cancer cells by inhibiting platelet aggregation (33, 34).

During the past 3 decades, studies have shown that regular, low doses (75 to 300 mg) of aspirin reduce the risk of cancer in the general population, with a significant benefit (35). Since 1988, many studies have demonstrated the positive effect of aspirin on colorectal cancer (36, 37). Recent studies have shown that the risk of colorectal cancer is significantly reduced after using aspirin continuously for 5 years and that the protective effect persists at 20 years of follow-up, and a longer duration of aspirin use is related to higher protection (38, 39). Similarly, aspirin may reduce deaths from prostate, biliary, and liver cancers (40–42). However, studies have also suggested that aspirin may accelerate cancer progression in people over 70 years because of its bleeding risk (32, 43). Therefore, aspirin should be used carefully in population with high risk of bleeding, while long-term use is recommended for those at high risk of CVD.

### Statins

Statins can reduce blood cholesterol levels by inhibiting the rate-limiting enzyme of the MVA metabolic pathway, namely HMG-CoA reductase (HMGCR), which can significantly reduce morbidity and mortality due to CVD. Meanwhile, statins also inhibit the transport of receptors on the surface of cell membranes, thereby reducing cancer cell growth, survival, migration, metastasis, inflammation, angiogenesis, promoting apoptosis, and having a protective effect on tumors. Statins may also have an anti-cancer effect by depleting cholesterol in certain situations. Lipophilic statins are more effective in inhibiting viral replication, enhancing therapeutic effectiveness, and passively entering the cell membrane, providing a more sustained and effective cholesterol-dependent anti-HCC effect (44, 45).

Previous studies have shown that statins use is associated with a 13–40% reduction in the incidence of cholangiocarcinoma (46, 47). In a large clinical case analysis, statins are associated with a 25% reduction in the risk of extrahepatic cholangiocarcinoma and improved survival for patients with distal cholangiocarcinoma. Moreover, the risk decreases with the duration of statin use (48). A previous study has suggested that statins may prevent cholangiocarcinoma, but due to the low incidence of cholangiocarcinoma, the association between statins and cholangiocarcinoma still needs to be further verified (49). A recent Swedish viral hepatitis cohort study reported a dose-dependent and time-dependent reduction in the risk of liver cancer, all-cause mortality and liver-related mortality in patients with viral hepatitis treated with lipophilic statins (50). Many studies have shown that statins are associated with a lower incidence of colon cancer. Statins also reduce the risk of progression from non-advanced adenomas to colon cancer, especially proximal lesions, and prevent colorectal cancer recurrence after treatment (51). Additionally, inhibition of the HMG-CoA reductase gene is associated with a lower incidence of epithelial ovarian cancer. However, the effect of statins on ovarian cancer has not been determined and needs to be further investigated (52). Furthermore, retrospective studies have shown that breast cancer patients receiving both statins and anthracyclines have a lower risk of heart failure than those who do not receive statins; however, the difference is not significant in breast cancer patients receiving trastuzumab (53).

### Antihypertensive Drugs

According to statistics, about 37% of cancer patients have hypertension, and the active and effective control of blood pressure by antihypertensive drugs can prevent the occurrence of cardiovascular complications and improve the quality of life in cancer patients (54). At present, the widely using antihypertensive drugs include ACEIs, angiotensin-receptor antagonists (ARBs), β-blockers, calcium antagonists, diuretics, α-blockers and central sympatholytic drugs. The relationship between antihypertensive drugs and cancer has received widespread attention recently, and the interaction between several antihypertensive drugs and malignant tumors is still unclear. A 1,998 article published in The Lancet studied the patients who used blood pressure drugs for more than 3 years. Patients who used ACE inhibitors had the lowest relative risk of developing cancer, while patients who used calcium channel blockers (CCBs), diuretics, and beta-blockers, had no significant effect on cancer risk (55). In recent years, studies have shown that various antihypertensive drugs may be associated with cancer, while renin-angiotensin system inhibitors (RAS inhibitors) may have a more comprehensive protective effect (Table 1).

**Table 1:** Impact of commonly used antihypertensive drugs on cancer risk.

**Antihypertensive drugs**	**Effects on various cancers**
Diuretics	Increase the risk of breast and skin cancer; may increase the incidence of urinary cancer
CCBs^a^	Increase the incidence of skin cancer and urinary system cancer
β2 receptor blocker	Reduce the incidence of lung cancer and digestive system tumors, and have different conclusions on the effects of head and neck squamous cell carcinoma, breast cancer, skin cancer and urinary system tumors.
ACEI/ARB^b^	Reduce the incidence of breast cancer, urinary tract cancer, and digestive tract cancer May increase the incidence of lung cancer, especially after using high dose ACEI (56).

a *CCBs, Calcium channel blockers*;

b *ACEI, angiotensin-converting enzyme inhibitors; ARB, angiotensin-receptor antagonists*.

#### Diuretics

Certain diuretics (such as thiazide diuretics), are considered photosensitive drugs and can increase the risk of skin cancer associated with ultraviolet (UV) light by damaging DNA (57). Thiazide diuretics are associated with insulin resistance, a recognized risk factor for breast cancer (58).

Two large studies from Denmark and Iceland have shown that hydrochlorothiazide is significantly associated with an increased risk of skin cancer, possibly in a dose-dependent relationship (59, 60). However, other studies have shown no significant relationship between hydrochlorothiazide and the risk of skin cancer, which may be due to individual sensitivity to UV light, which led to different results (61). Since 1980, many studies have shown that diuretics are positively correlated with the risk of breast cancer. The incidence of breast cancer increases by 16% in using diuretics for more than 10 years, and the use of diuretics is highly correlated with the poor prognosis of breast cancer patients (62). A case-control study of hypertensive and non-hypertensive patients on antihypertensive drugs showed that methotrexate, thiazide, and loop diuretics increased the risk of renal cell carcinoma by 40%, with women at a higher risk than men (63, 64). However, high blood pressure itself can cause kidney damage, so more clinical studies are needed to confirm this statement.

#### Calcium Channel Blockers

To date, available data suggest that CCBs increase the tumors incidence by inhibiting apoptosis or interfering with cell differentiation through calcium triggering signals. Moreover, CCBs reduce intracellular calcium levels and impair the process of programmed cell death. The body is prevented from destroying damaged cells to prevent malignancy, leaving them to replicate when desired (65, 66).

A meta-analysis combining 11 related studies showed that long-term (>9 years) treatment with CCBs increased the incidence of malignancy (67). Rothschild's large population-based study and meta-analysis showed a slightly increased risk of lung and prostate cancer in calcium antagonist users, both in a time-dependent manner (68–70). CCBs were associated with a 1.6-fold increased risk of breast cancer in those who used CCBs for more than 2 years, especially invasive ductal carcinoma and invasive lobular carcinoma of the breast thant hose who had never used antihypertensive drugs (71, 72).

#### Beta-Receptor Blockers

In recent years, several studies have found that selective beta 2 blockers (β2 blockers) may reduce the recurrence and metastasis of cancer and thus increase overall survival in cancer patients. Selective beta 1 blockers have been shown to have no beneficial effect on cancer (73). Epinephrine and norepinephrine can induce tumor cell invasion and migration, thereby affecting lymph node invasion and metastasis, and this effect is mediated by the β-adrenergic pathway, especially the β2 receptors (74–77). Therefore, beta-blockers compete with epinephrine and norepinephrine for effective beta-adrenergic receptors to reduce the migratory activity of cancer cells and can also alter tumor growth, invasion, apoptosis, and angiogenesis to prevent tumor metastasis (78). Furthermore, the selective β2 receptor blocker propranolol can reduce the expression of the proliferative antigen Ki-67 and increase the phosphorylation of the tumor suppressor gene *P53* in early breast cancer, thus slowing down cell proliferation and inducing cell apoptosis, which has a positive effect on breast cancer pateints (79).

Barron suggested that women who took propranolol in the years before breast cancer diagnosis were significantly less likely to develop T4 tumors in a large population study, with positive lymph nodes (N2/N3), or metastases and significantly lower mortality rate than women who did not take propranolol. Besides, prolonged use of propranolol may reduce T4 tumorigenicity (73). Moreover, propranolol has a protective effect on head and neck cancer, stomach, colon, and prostate cancer, especially when used for more than 1,000 days (46). Inhibition of angiogenesis reduces bacterial translocation; thus, non-selective beta-blockers have been shown to reduce the incidence of liver cancer in patients with cirrhosis (80).

#### ACEI/ARB

RAS is well known for its control over the body's internal environment stability. Recently, there have been many studies reported RAS involvement in the complex carcinogenic mechanism. It is associated with proliferation signaling, resistance to cell death, induction of angiogenesis, energy metabolism reprogramming, inflammation, cell migration, invasion, and metastasis, thereby promoting vascular endothelial growth factor-mediated angiogenesis in malignant tumors and increasing proliferation of malignant tumor cells (81, 82). Therefore, using drugs that inhibit the RAS (mainly ACEI and ARB) can slow the rate of tumor growth. Furthermore, the risk of most cancers also decreases by using for long time (83).

A recent retrospective study of 73,170 patients with breast cancer patients using ARB improved patient's survival rate and reduced mortality. Patients who used other antihypertensive drugs also had reduced mortality, but cannot rule out it is due to blood pressure control or have positive effects on cancer (84). Similarly, compared with other antihypertensive drugs, patients on ACEI have a lower incidence of prostate cancer; hence, ACEI may improve their survival rate (85, 86). RAS inhibitors slow the progression of gastrointestinal cancer. Using ACEI over 3 years was associated with a 29% reduction in the risk of esophageal adenocarcinoma, and high daily doses were associated with a 45% risk reduction (87). RAS inhibitors have been linked to a protective effect against pancreatic cancer, with a 39% risk reduction after 1–3 years of use, but no significant effect on long-term use (88). A case-control study of patients with hypertension suggested that RAS inhibitors reduced the incidence of colon cancer, with long-term use decreasing the risk by 16 and a 25% reduction after 5 years of use. The greater the dose, the more significant the positive effect (89). The dose of ACEI is inversely proportional to the size of adenomatous polyps.

However, ACEI increases the incidence of lung cancer in patients with hypertension. The existing research points out that bradykinin (BK, a 9-peptide substance with cardioprotective effects) was found in lung cancer tissue and substance P. Many tumor cells expressed higher levels of BK and the related receptors that directly release vascular endothelial growth factor that stimulate the growth of cancer cells and angiogenesis, leading to increased risk of lung cancer. Additionally, ACEI promotes the buildup of these two chemicals in the lungs (90, 91).

Hicks et al. (92) in 2018 conducted a study, which included more than 90,000 patients with hypertension, was followed for 13 years to compare ACEI use and lung cancer incidence. This study confirmed that ACEIs could lead to an increased incidence of lung cancer. Lin et al. (93) further compared lung cancer incidence in hypertensive patients using ACEIs vs. ARBs and found that lung cancer incidence was significantly higher in patients using ACEIs than in those using ARBs. Moreover, they found that the higher the dose and longer the duration of ACEI use, the higher the incidence of lung cancer. Kumar et al. (94) and Hsu et al. (95) subsequent studies support this view. There are different conclusions, a 2021 study by Lee et al. (96) concluded that there was no significant difference in the effect of ACEI and ARB on lung cancer. Similarly, a meta-analysis, enrolled 13 observational studies with 458,686 ACEI users, conducted by Batais et al. (97) suggested that ACEIs were not associated with an increased risk of lung cancer. Although most studies reported a negative effect of ACEIs on lung cancer, more prospective studies are needed to confirm the effect of antihypertensive drugs on cancer incidence and progression.

Different cancer stages or different types of cancer have different responses to RAS blockers. As the only antihypertensive drugs that have definite effects on cancer, RAS blockers should be used carefully in clinical practice to achieve treatment optimization.

### Other Drugs

A retrospective study suggested that in patients with atrial fibrillation (AF), oral anticoagulants, gastrointestinal bleeding, urogenital bleeding, and bronchopulmonary bleeding often increased the risk of cancer, and there is a strong correlation with the severity of bleeding. It is worth mentioning that this new cancer is often detected within 6 months after bleeding, but this may be related to more frequent follow-ups. In any case, patients with AF receiving oral anticoagulants should be alert to the occurrence of cancer once they have bleeding in the above-mentioned organs (98).

## Early-Onset and High-Incidence Cardiovascular Risks of Cancer Patients and Their Management Strategies

### Cancer Treatment Is Prone to Cardiovascular Disease

Cardiotoxicity due to antineoplastic therapy is induced by multiple factors, mainly including oxidative stress (99) [OS; including mitochondrial functional impairment (100), myocardial apoptosis (101, 102)], microtubule dysfunction (103, 104), and disruption of myocardial immune homeostasis (105, 106).

OS refers to an imbalance in the body's oxidative and antioxidant systems that tend toward oxidation, causing abnormalities in the body's biochemical and physiological processes and damaging endothelial tissue (107). Oxidants of oxidative stress refer to reactive oxygen species (ROS) or nitrogen substances (RNS) as well as free radicals. The direct effects of both inflammation and ROS are mediated by the activation of macrophages in the arterial wall (108). Neutrophils and monocytes/macrophages are the main sources of ROS, and oxidative stress increases the production of chemokines (MCP-1, CSF-1) and adhesion molecules (ICAM-1), tending to shift the redox balance toward a peroxidized state by promoting the aggregation of these cells (109). Since the heart has a weak antioxidant capacity (102), high concentrations of ROS predispose cardiomyocytes to mitochondrial damage and lipid peroxidation, affecting myocardial function. High oxidative status in elderly patients could explain the high incidence of cancer in elderly patients; therefore, chronic inflammation and oxidative stress should also be considered risk factors for cancer in the elderly (110). Drugs that mediate cardiotoxicity through oxidative stress are mainly anthracyclines and anti-epidermal growth factor receptor 2 (ErbB2) drugs, which increase the production of ROS and RNS, inhibit oxidative phosphorylation (111), lead to mitochondrial damage in cardiomyocytes (110), and ultimately result in irreversible myocardial damage. Mitochondria are important target tissues, and patients receiving tyrosine kinase inhibitors (TKI) can also suffer from cardiac complications due to impaired mitochondrial function that lead to cell death (106). Paclitaxel alters the process of cell division by affecting microtubule function, and it also affects the level of histamine in the body and stimulates the development of cardiotoxicity (106). Notably, immune checkpoint inhibitors (e.g., PD-1) mainly affect immune regulation, i.e., they influence T-cell effector function by inhibiting T-cell downstream signaling and hinder the immune organ from fighting against cancer cells (112, 113). Therefore, immune checkpoint inhibitors have been used in solid and hematological cancers to enhance the immune system's potential to fight cancer cells (114). Although existing studies suggest that immune checkpoint inhibitors are less likely to be cardiotoxic, immunotherapy may cause life-threatening events in patients (cardiac arrest, fulminant myocarditis, shock) by disrupting immune homeostasis in the myocardium (115, 116).

#### Cardiac Dysfunction

In patients with antineoplastic therapy-induced congestive heart failure, a reduction in LVEF of more than 10% and <50% is diagnostic of cancer therapy-induced cardiac insufficiency (cancer therapy drug-associated cardiac insufficiency/CTRCD). CTRCD usually appears months to years after treatment and is reversible in 75% of patients after withdrawal; however, it may affect long-term prognosis in 25% of patients, especially in patients with left bundle branch block. Most of these patients have no obvious symptoms, left ventricular dysfunction was diagnosed in some patients, while only a small number of patients develop symptomatic heart failure (117).

The most common anti-cancer drug that causes heart failure is anthracycline, the main treatment drug for many lymphomas, soft tissue sarcomas, and breast cancer, causing about 43% of those at risk (118). The main mechanism is the irreversible damage of cardiomyocytes with a high density of mitochondria, which induces myocardial remodeling and leads to cardiomyopathy (119). This is followed by TKI and immunotherapy, but the damage to the heart muscle is temporary and reversible. Previous studies suggested that the targeted drug trastuzumab may also increase the risk of cardiomyopathy by four times, and that when combined with anthracyclines, the risk increases by seven times (120, 121).

Women receiving anthracyclines are less likely than men to have cardiac insufficiency secondary to chemotherapy (122). The mechanism of cardiac insufficiency caused by anthracyclines is mainly due to oxidative stress-mediated oxidative damage in cardiomyocytes and abnormal mitochondrial function (123), which is less reported in female individuals (122). Notably, the occurrence of cardiac insufficiency complications in pediatric cancer patients does not appear to be significantly correlated with gender, as pediatric and postmenopausal females are more likely to develop cardiac insufficiency from antineoplastic therapy, suggesting that estrogen modulates abnormal oxidative stress in cancer patients receiving anthracyclines (124, 125). Estrogen enhances myocardial resistance to ischemia/reperfusion injury, i.e., attenuates abnormal oxidative stress and apoptosis (126). Thus, estrogens are cardioprotective. Sex differences in cardiotoxicity are not only present in anthracycline treatment but also cardiotoxicity caused by paclitaxel and tyrosine kinase inhibitors. The cardiotoxicity produced by different drugs differed concerning age and gender (Table 2). Sex-related genes are also involved in regulating the development of chemotherapy-related cardiac insufficiency (139). However, there are few studies related to the effect of gender on the efficacy and complications of antitumor therapy, and more gender- and age-specific studies are needed in the future to clarify the effectiveness of antitumor therapy and provide more theoretical support for clinical use.

**Table 2:** Differences in cardiotoxicity due to antineoplastic drugs.

**Antiblastic drugs**	**Major symptoms**	**Age differences**	**Gender differences**
Anthracyclines	Cardiac insufficiency	No age difference in the development of congestive heart failure (CHF) in metastatic breast cancer patients >40 years of age treated with adriamycin (127). Increased incidence of CHF in patients older than 65 years of age with breast or lung cancer treated with adriamycin compared to those younger than 65 years of age (128). Age >65 years in patients with hematologic tumors treated with adriamycin may be a risk factor for the development of HF (129).	Pediatric patients-greater cardiovascular risk in women (124, 125, 130). Adults-greater reduction of LVEF in men (131). Adult-High incidence of cardiogenic adverse events in men (132).
Tyrosine kinase inhibitors (TKI)	AF and hypertension (133)	Patients receiving TKI are more likely to experience cardiotoxicity as they get older (134).	Sunitinib-more is likely to develop cardiotoxicity in women (135). Other drugs in TKI, such as imatinib and sorafenib-no gender difference in cardiotoxicity (136).
Paclitaxel	Bradycardia and coronary artery spasm	-	Women are more sensitive to paclitaxel treatment and are less likely to experience cardiotoxicity (137).

*Anthracyclines are the most prominent antineoplastic agents for inducing cardiotoxicity. Cardiotoxicity is age and gender-related, as well as being dose-dependent (128, 138)*.

#### Coronary Artery Disease (Acute Vasospasm and Atherosclerosis)

With the increasing number of cancer survivors, vascular toxicity has become the second most widely concerned disease after cardiac toxicity. In addition to the high incidence of venous thromboembolism, arterial toxicity has also attracted attention due to the increase of cancer patients, the prolonged life span, and the continuous progress of cancer treatment. A case study of children's study points out that had not been treated for cancer patients with systemic inflammation and increased risk of atherosclerosis, and a diagnosis of age is smaller, the higher their risk of dying from heart disease, 2 years after another child study, points out that accepting radiotherapy or chemotherapy and survival over the age of 35 patients compared with a normal person five times the risk of myocardial infarction (AMI) (140, 141). However, the overall incidence of myocardial infarction is not high. Arterial thromboembolism is also common in pancreatic, gastric, and lung cancer patients, like venous thromboembolism. Among all patients with vascular complications, lung cancer patients have the highest mortality rate and colon cancer patients have the highest bleeding risk (142, 143). Patients with metastatic cancer complicated with acute myocardial infarction have a poor prognosis, and the cause of death is usually associated with hemorrhage and reinfarction (144).

Ischemic heart disease is critical cardiotoxicity in patients treated with 5-fluorouracil during antitumor therapy. Vascular endothelial growth factor inhibitors can cause coronary spasms and even acute myocardial infarction due to endotheliotropic and erosion of monolayer vascular endothelial cells (145, 146). Paclitaxel can cause acute coronary spasms and even myocardial infarction (147). In such patients, the ST segment elevation rapidly reduces after immediate administration of nitrates. Due to its endothelial toxicity, which is not easy to eliminate, cisplatin can also cause coronary artery diseases, and the toxicity increases with the increase of its dose (148). Existing findings show that radiation therapy can change vascular reactivity and vascular spasm and possible acute severe endothelial injury and acute myocardial infarction (MI). The prolonged radiation exposure time is proportional to the risk of myocardial infarction. Due to the cancer patients own existence of cardiovascular risk factors and the role of anti-cancer treatment, prolonged endothelial cells to rebuild (149, 150). Protecting the heart from radiation may significantly reduce the risk of coronary atherosclerotic heart disease (151).

#### Arrhythmias

Various arrhythmias may occur in cancer patients during anti-cancer treatment. Inflammatory infiltration of the heart may result in pericarditis or cardiomyopathy that involves the cardiac conduction system and results in the atrioventricular block, prolonged QT interval, and AF. Radiotherapy causes radiation injury and promotes myocardial fibrosis, resulting in an atrioventricular block and AF, but rarely causes ventricular arrhythmias (152), so it may become an alternative therapy for invasive ventricular ablation of ventricular arrhythmias. The arrhythmias induced by antitumor therapy may also be related to drug interactions, drug accumulation, and electrolyte disturbance. The common arrhythmias in cancer patients mainly include AF, prolonged QT interval, ventricular arrhythmias, and cardiac arrest (152).

In 1993, the first arrhythmia caused by antineoplastic therapy was proposed, about 30% of patients who used chemotherapy drug paclitaxel developed asymptomatic bradycardia (104). Later studies suggested that thalidomide, palazonib, sunitinib, and crizotinib may also cause bradycardia. About 72% of patients treated with the chemothermic arsenic trioxide extended their QT interval from baseline by more than 30 ms, with half of those exceeding 60 ms (153). Many targeted drugs cause prolongation of the QT interval (154, 155). Generally, the prolonged QT interval resolves gradually as the drugs are metabolized. However, if the upper limit is exceeded, antitumor drugs should be discontinued to avoid torsade de points ventricular tachycardia. AF is closely linked to cancer, and they share the same risk factors: obesity and inflammation. A study showed a 20% increase in the incidence of cancer within 1 year of AF onset. AF occurred as a cardiotoxic complication of antitumor therapy with anthracycline, ibrutinib, melphalan, and paclitaxel (156, 157).

#### Thrombotic Disease and Peripheral Vascular Disease

Venous thromboembolism (VTE), which includes deep vein thrombosis (DVT) and pulmonary embolism (PE), is second only to disease progression as the leading cause of death in cancer patients. Pulmonary embolism occurred in half of untreated DVT patients. About one-third of untreated pulmonary embolism patients die, most of whom have recurrent thromboembolism (158, 159). Compared with normal people, the incidence of VTE in cancer patients is at least 4 times higher, and a higher risk of VTE often indicates a poor prognosis of the cancer patients. A previous study showed that cancer of the pancreas, bile duct, and liver is associated with a higher risk of VTE (160).

Cancer patients release pro-inflammatory factors and pro-coagulation active substances, thereby promoting the adhesion between blood cells and blood vessels, resulting in a high coagulation state. The main factors of cancer patients that predispose to VTE include the type of cancer, central venous catheter chemotherapy, radiotherapy, surgical treatment, and related drug side effects (161).

#### Others: Hypertension, Valvular Heart Disease, Pericardial Disease

Hypertension is closely related to the occurrence of tumors. High blood pressure and cancer have some common risk factors (age, active or passive smoking, diabetes, dyslipidemia, overweight or obesity, low physical activity, unhealthy diet) (162). Vascular endothelial growth factor (VEGF) possibly plays an important role in the pathogenesis of hypertension and tumor by stimulating angiogenesis (163, 164). Forty-one years after Judah Folkman (165) proposed that tumor growth depends on the formation of new blood vessels by secreting factors, hyperactive angiogenesis is now becoming a therapeutic target for cancer (166). However, given the common biological characteristics of tumors and hypertension, some anti-tumor drugs can increase the incidence of hypertension. At present, it is believed that the anti-tumor drugs that can cause hypertension mainly refer to VEGF signal inhibitors, with a 19–47% incidence of hypertension (167). VEGF signal inhibitors may lead to an imbalance between vasodilators and vasoconstrictors, loss of capillary microcirculation, and altered glomerular function, all of which contribute to hypertension (168, 169). Some scholars believe that hypertension may be related to the effectiveness of anti-cancer treatment. Tanaka et al. (170) found that the development of hypertension in the early stage of treatment is related to the anti-tumor effect and maybe a predictor of treatment effect.

Anti-tumor therapy-related valvular heart disease is mainly caused by radiotherapy, especially involving the left heart valve. The pathological manifestations were valve tip and leaflet thickening, calcification, and retraction. Similarly, radiotherapy over 2 years can lead to pericarditis in up to 20% of tumor patients, so it is recommended that radiotherapy doses of <10 Gy should be limited to patients without prior cardiac disease during radiotherapy for thoracic tumors (171).

Cardiac metabolic syndrome is a condition caused by various metabolic disorders that affect about one in four adults. Saxena et al. (172) proposed that cardiac metabolic syndrome is associated with various cancers, especially pancreatic and rectal cancer in females and prostate cancer in males.

### Cancer and Cardiovascular Disease Prevention

Poor lifestyle is a common risk factor for CVD and cancer, while a low-risk lifestyle reduces the incidence of cancer, CVD and diabetes, as well as the mortality due to related diseases, and prolongs the life expectancy of healthy people. A low-risk lifestyle includes non-smoking, a BMI of 18.5–24.9 kg/m^2^, moderate daily exercise (≥30 min/day), moderate alcohol consumption (5–15g/day for women, 5–30g/day for men), and a high-quality diet (173). Moderate drinking may prevent CVD, but cancer risk is relatively increasing, so non-alcoholic drinkers are not recommended to start drinking to prevent CVD (173). Dietary supplements of omega-3 fatty acids or vitamin D to prevent cancer and CVD are also not recommended for the general population, as they may cause problems with existing health conditions (174).

Prior to cancer treatment, especially before using treatment measures known to have cardiovascular toxicity, patients should be screened for the risk of underlying CVD, diabetes, and other related diseases through Electrocardiogram (ECG), echocardiography, biomarkers, and other tests. Heart rate variability (HRV) may predict cardiovascular complications in breast cancer (175). More elaborate screening tests for underlying diseases and comorbid conditions should be undertaken for patients with pre-existing CVD. For patients with tumors at high cardiovascular risk, close monitoring of relevant indicators is recommended and antitumor therapies with clear cardiovascular toxicity should be avoided.

### Recommended Diagnostic Methods

#### Blood Pressure Monitoring and Electrocardiogram

The diagnostic criteria for hypertension in cancer patients are the same as those in the general population.

The diagnosis of arrhythmia and acute ST-segment elevation myocardial infarction in cancer survivors can be confirmed by paying close attention to the dynamic changes of ECG. For paroxysmal arrhythmia, a dynamic ECG is feasible to make a clear diagnosis. Aggressive electrophysiological tests can be used to diagnose suspected arrhythmias, but the necessity of these tests depends on the patient's general state and life expectancy (176).

#### Imaging Examination

Echocardiography is the first choice for cancer patients to monitor cardiac function (i.e., LVEF) and diagnose valvular heart disease. 3D echocardiography is recommended as the first choice so that the endocardial boundary can be seen more clearly (177). Cardiac magnetic resonance imaging (CMR) has become the clinical gold standard for measuring left ventricular volume and ejection fraction, followed by radionuclide ventricular angiography (RVG)/ multigate cardiac pool imaging (multigate acquisition, MUGA). An endomyocardial biopsy can determine the extent of myocardial injury in cancer patients, but due to its invasive nature, the diagnosis is usually confirmed by the patient's symptoms and imaging examination (178, 179).

Arterial and venous ultrasound of bilateral lower extremities is preferred for VTE diagnosis. Computed tomography pulmonary angiography (CTPA) is recommended to confirm PE after DVT is diagnosed. CTPA is the preferred imaging modality for diagnosing PE. When patients have symptoms highly suspicion of PE, such as dyspnea, chest pain, hemoptysis, and cough, accompanied by hypoxemia, along with DVT, as suggested by arterial and venous ultrasound of lower limbs, the CTPA should be performed timely to make a confirmed diagnosis. CTPA is contraindicated in patients with contrast hypersensitivity, renal insufficiency, hypotension, advanced heart failure, or unable to perform CT scanning due to complex comorbid conditions or difficulty lying flat (159).

#### Biomarker

Biomarkers play an important role in the prevention and diagnosis of CVD (Table 3). However, a single biomarker has certain limitations, and many factors can lead to abnormal results, so a definite diagnosis generally requires imaging findings and lab tests.

**Table 3:** The significance of monitoring biomarkers in cancer patients.

	**Clinical significance**
NPs^a^	NPs can be used as an early biomarker for cardiac insufficiency caused by conventional chemotherapy. However, there is no clear evidence for the diagnosis of cardiac insufficiency caused by other antitumor therapies.
D-D^b^	Although a definite diagnosis of VTE^d^ cannot be made, higher serum D-dimer levels in cancer patients may predict an increased risk of mortality due to cancer and coronary heart disease (180). Oikawa (181) recently proposed that D-D can be used as an important parameter to predict cardiac dysfunction in cancer patients, with a cut-off value of 1.65μg/ ml.
cTn^c^	In patients receiving trastuzumab or high-dose chemotherapy, increased cTn indicates abnormal heart function and poor prognosis.

a *NPs mainly refer to BNP and NT-proBNP in the monitoring indicators of cardiac insufficiency, where BNP > 100 pg/ml indicates cardiac insufficiency, and NT-proBNP <125 ng/L can be used as an exclusion criterion*;

b *D-D, D-dimer*;

c *cTn, cardiac troponin*;

d *VTE, Venous thromboembolism*.

##### Natriuretic Peptide

Nearly half of the patients with cardiac dysfunction are not accompanied by reduced LVEF, so when patients have overt dyspnea, but no history of myocardial infarction and signs of pulmonary edema, it is recommended to measure natriuretic peptide (NP): B-type natriuretic peptide (BNP) or N-terminal precursor B-type brain natriuretic peptide (NT-proBNP) level (182).

##### D-Dimer

D-dimer (D-D) is the first choice for VTE diagnosis in a normal population. However, given the higher pathophysiological coagulation tendency in cancer patients, increased plasma D-D level is generally found in cancer patients. Therefore, arterial and venous color Doppler of the bilateral lower extremity is considered the first choice for VTE diagnosis in cancer patients (183).

##### Cardiac Troponin

The diagnostic approach of coronary heart disease in cancer patients is the same as that in normal people. Cardiac injury markers include creatine kinase MB (CK-MB), myoglobin, and cardiac troponin (cTn); among them, cTn is considered more important. For early reinfarction in patients with MI, CK-MB is relatively more significant (184).

#### Coronary Angiography

In addition to the patient's symptoms and vital signs, coronary angiography is the gold standard for diagnosing coronary artery disease (CAD) and evaluating vascular status. Diagnosis of CAD can be challenging in cancer patients, and some of the anti-tumor drugs mentioned earlier can cause transient coronary spasms that mimic the symptoms of a heart attack.

For patients at low risk for CAD, CCTA can be considered. Indications for coronary angiography are that the patient is suitable for coronary revascularization and that acute coronary syndrome or angina is not adequately controlled by optimal medication (185).

### Early Detection and Treatment of Cardiovascular Diseases in Cancer Patients

When the detection time and treatment time for complications of cardiac insufficiency in cancer patients are doubled, the chance of LVEF returning to normal will be reduced by 25% (186). Therefore, cardiovascular complications in cancer patients should be strengthened (Table 4). First, it should be clear whether the patient is a patient with a high risk of CVD such as hypertension, diabetes, smoking, alcohol consumption, hyperlipidemia, obesity, poor lifestyle and presence of a family history of heart disease, and a 12-lead ECG should be routinely checked before anti-tumor treatment. Blood pressure and myocardial enzymes and other indicators should be recorded. Given that the electrolyte imbalance, abnormal thyroid function, or renal function may cause arrhythmia (193), patients should be routinely checked before cardiovascular toxicity occurs, and changes in the patient's ECG and myocardial enzymes should be monitored during anti-tumor treatment. Some drugs for treating tumor comorbidities, such as antiemetics and psychotropic drugs, can prolong the DT interval. It should be clear whether the arrhythmia caused by antitumor therapy or the treatment of comorbid drugs is caused. In high-risk situations, it is recommended to switch to other drugs to treat comorbidities (186).

**Table 4:** Prevention and treatment of cancer patients with cardiovascular disease.

	**Prevention and evaluation before cardiovascular complications**	**Treatment of cardiovascular complications in cancer patients**	**Clinical advice**
Cardiac dysfunction	During the anti-tumor treatment, assessment should be conducted at least every 3 months, and monitoring should be conducted at least every 6 months for 2 years after the completion of treatment. For patients with pre-existing cardiac insufficiency, it is recommended to monitor once a month.	Beta-blockers and ACEI/ARB should be used as early as possible, while other conventional therapies, such as diuretics and cardiac, should be used as appropriate in conjunction with the patient's symptoms. For most patients with cardiotoxicity, especially patients with left bundle branch block and heart failure, cardiac resynchronization therapy may relieve symptoms and reverse ventricular remodeling. However, ventricular assist devices are generally not recommended.	For advanced heart failure, heart resynchronization therapy and heart transplantation may produce higher returns in addition to drug treatment.
Coronary artery disease	Prevention of arterial disease should start with endothelial health, including statins, angiotensin-converting enzyme inhibitors and active exercise (187). Aspirin can be used as the main preventive drug to reduce the occurrence of arterial embolism and the progression of atherosclerotic plaque.	It is recommended to evaluate the severity of the patient's arterial toxicity and then determine whether to continue anti-tumor therapy. The first choice for treating patients with vasospasm is vasodilators, such as nitrates and calcium channel antagonists. When cancer patients are combined with atherosclerosis, drug therapy is the basis. The treatment measures mainly include adequate control of blood pressure and blood sugar, anti-platelet aggregation and lowering blood lipids, stabilizing plaque, slowing down disease progression, and eliminating the cause of myocardial infarction. Combining anticoagulation and interventional therapy may bring longer survival time to cancer patients with myocardial infarction. Drug-eluting stents (DES) are recommended for patients undergoing coronary stent implantation (143).	Patients who have received coronary revascularization and have a good prognosis can be given cancer treatment based on the benefit of the patient, but aspirin, calcium channel blockers and long-acting nitrate drugs should be given 3 days before the drug, and the ECG should be monitored continuously, and once symptoms such as angina pectoris appear again, treatment should be stopped immediately.
Arrhythmia	Re-check the patient's electrolytes, thyroid function and renal function within 7–15 days after treatment and after each treatment plan change, and should be monitored monthly for the first 3 months of treatment. People taking the chemical arsenic trioxide should monitor their ECG at least weekly.	Beta-blockers (atenolol and metoprolol) are the drugs of choice for controlling ventricular rate to treat atrial fibrillation. Non-dihydropyridine-calcium channel blockers are also optional but must be used appropriately according to the patient's heart condition. Cardioversion can be considered, when necessary, but patients who use ibrutinib are more likely to relapse after cardioversion. At the same time, amiodarone and digoxinine interact with certain cancer treatment drugs and should be used with caution. For patients with symptomatic or reduced ejection fraction heart failure and atrial fibrillation, radiofrequency ablation is also a necessary option (152). The anticoagulation strategy for cancers with atrial fibrillation is still based on the CHA2D2-VASC score. However, anticoagulant therapy may not be effective in the hypercoagulable state of cancer. Low molecular weight heparin (LMWH) is the first choice for anticoagulation therapy, followed by oral anticoagulants (DOAC).	-
Thrombotic disease and peripheral vascular disease	The use of anticoagulants for primary prevention of cancer patients is generally not recommended, but patients undergoing major cancer surgery should receive prophylaxis at least 7 days before surgery (188). Patients with a Khorana score ≥ 3 or Khorana score ≥ 2 and a high risk of thrombosis can start primary preventive anticoagulation therapy.	All cancer patients with new or recurrent VTE require anticoagulation therapy, and it is recommended to continue anticoagulation therapy for at least 3–6 months. LMWH or edoxaban is the first anticoagulant choice, but there may be technical limitations or patient intolerance. Now you can use LMWH or the oral anticoagulant edoxaban for 5–10 days, and then use DOAC other than warfarin or edoxaban. If active cancer or recurrent VTE occurs under active treatment, systemic treatment should be continued (143). The inferior vena cava filter (IVC) is used for VTE patients with contraindications to anticoagulation, and clinically, it is also used for patients with active anticoagulation therapy but still relapsed VTE^a^.	Before using IVC, the patient's willingness and life expectancy should be evaluated, and it is generally not the first choice for VTE cancer patients.
Others: hypertension, valvular heart disease, pericardium disease	The 2018 ESC/European Society of Hypertension (ESH) Arterial Hypertension Management Guidelines recommend that blood pressure be monitored once a week during the first cycle of cancer treatment, and at least once every 2–3 weeks thereafter (189). Antihypertensive therapy helps maintain the treatment plan and reduce the risk of serious complications, including malignant hypertension and reversible posterior leukoencephalopathy.	Patients with hypertension (≥140/90 mmHg) or elevated diastolic blood pressure (≥20 mmHg) should receive ACE inhibitors, ARBs, calcium channel blockers, or combination therapy. The calcium channel blockers diltiazem and verapamil should be avoided. Since VEGF inhibitors may cause diarrhea and dehydration, electrolyte disturbances caused by diuretics may aggravate, diuretics should be used with caution (190). For moderate hypertension (systolic blood pressure > 160 mmHg, diastolic blood pressure BBB > 0 mmHg), anti-tumor therapy should be suspended and antihypertensive therapy should be given until the blood pressure returns to the pre-treatment level or below 150/100 mmHg, and the treatment can be resumed. Catheter valve implantation is recommended for valvular heart disease related to tumor treatment.	If hypertension is poorly controlled or a hypertensive crisis occurs after 1 month of treatment, anti-tumor angiogenesis drugs should be permanently discontinued. The blood pressure goal of cancer patients is <140/90 mmHg, and patients with diabetes or proteinuria can be reduced to <130/80 mmHg as appropriate.

a *For patients with venous thrombosis, LMWH is the first choice for anticoagulant drugs. DOAC becomes a secondary option due to the relatively high risk of major bleeding (191). Edoxaban is the preferred oral anticoagulant, but recent studies have shown that rivaroxaban may be the best choice (192). Patients with renal insufficiency should avoid LMWH and DOAC. It is recommended to use warfarin and monitor the INR during use*.

## Conclusion

Cancer survivors face several risks, including cancer recurrence and cardiovascular events. Cardio-Oncology provides a multidisciplinary approach to the entire treatment process and guides effective treatment of cancer patients with cardiovascular complications. However, the relevant studies are in their preliminary stages with several limitations. The positive results obtained from many studies may vary with age, gender, race, marital status, stage of cancer, time of diagnosis and surgical intervention. Therefore, large-scale study results are needed for further confirmation. Secondly, there is still much room to explore the interaction between cardiovascular drugs and anti-tumor drugs as well the effect of genes on the mechanism of these drugs. Clinical studies are required to decide the proper timing of discontinuation and restart of antitumor therapy due to cardiovascular complications and provide alternative therapies if needed.

When patients with both CVD and cancer are encountered clinically, the proper treatment strategies should be followed by clinicians. Moreover, oncologists should be informed of cardiovascular complications of antitumor therapy and their prevention, diagnosis and treatment. Furthermore, cardiologists should be alert to the high incidence of tumors caused by certain cardiovascular drugs in high-risk patients. Similarly, it calls on society to enhance the awareness and attention of cancer and CVD and hopes that doctors from all departments can cooperate to promote the continuous development of this discipline.

## Data Availability Statement

The original contributions presented in the study are included in the article/supplementary material, further inquiries can be directed to the corresponding authors.

## Author Contributions

YiW: reviewed the literature and drafted this review. YoW, XH, and CL: reviewed the literature, gave critical comments, and revised the manuscript. JS, BA, and JZ: gave critical comments and revised the manuscript. XM and ZC: reviewed the literature, gave critical comments, and revised the manuscript. All authors contributed to the article and approved the submitted version.

## Funding

The study was supported by the National Natural Science Foundation of China [YoW, Grant Number: 82170362], [CL, Grant Number: 82000347], and [JS, Grant Number: 81770374]. This study was also supported by Jilin Province Science and technology development plan project [YoW, Grant Number: 20190701066GH], [XM, Grant Number: 20210101246JC], China Postdoctoral Science Foundation [YoW, 2021M691209], and Jilin Medical and Health Talents Special [YoW, JLSWSRCZX2021-061].

## Conflict of Interest

The authors declare that the research was conducted in the absence of any commercial or financial relationships that could be construed as a potential conflict of interest.

## Publisher's Note

All claims expressed in this article are solely those of the authors and do not necessarily represent those of their affiliated organizations, or those of the publisher, the editors and the reviewers. Any product that may be evaluated in this article, or claim that may be made by its manufacturer, is not guaranteed or endorsed by the publisher.
